# Saturation effects and the concurrency hypothesis: Insights from an analytic model

**DOI:** 10.1371/journal.pone.0187938

**Published:** 2017-11-14

**Authors:** Joel C. Miller, Anja C. Slim

**Affiliations:** 1 Institute for Disease Modeling, Bellevue, WA, United States of America; 2 School of Mathematical Sciences, Monash University, Clayton, VIC, Australia; 3 School of Earth, Atmosphere, and the Environment, Monash University, Clayton, VIC, Australia; Universidad Nacional de Mar del Plata, ARGENTINA

## Abstract

Sexual partnerships that overlap in time (concurrent relationships) may play a significant role in the HIV epidemic, but the precise effect is unclear. We derive edge-based compartmental models of disease spread in idealized dynamic populations with and without concurrency to allow for an investigation of its effects. Our models assume that partnerships change in time and individuals enter and leave the at-risk population. Infected individuals transmit at a constant per-partnership rate to their susceptible partners. In our idealized populations we find regions of parameter space where the existence of concurrent partnerships leads to substantially faster growth and higher equilibrium levels, but also regions in which the existence of concurrent partnerships has very little impact on the growth or the equilibrium. Additionally we find mixed regimes in which concurrency significantly increases the early growth, but has little effect on the ultimate equilibrium level. Guided by model predictions, we discuss general conditions under which concurrent relationships would be expected to have large or small effects in real-world settings. Our observation that the impact of concurrency saturates suggests that concurrency-reducing interventions may be most effective in populations with low to moderate concurrency.

## Introduction

The HIV epidemic has had a significant impact worldwide, but especially so in sub-Saharan Africa [1]. The reasons for this difference are many, complex, and not fully understood [2, 3]. One proposed factor is a greater frequency of sexual partnerships that overlap in time, the so-called “concurrency hypothesis” [4, 5]. This hypothesis has received significant attention, but it is highly controversial (see for example [6–12] and [13–15]).

### Mechanisms of concurrency

**Fig 1:**
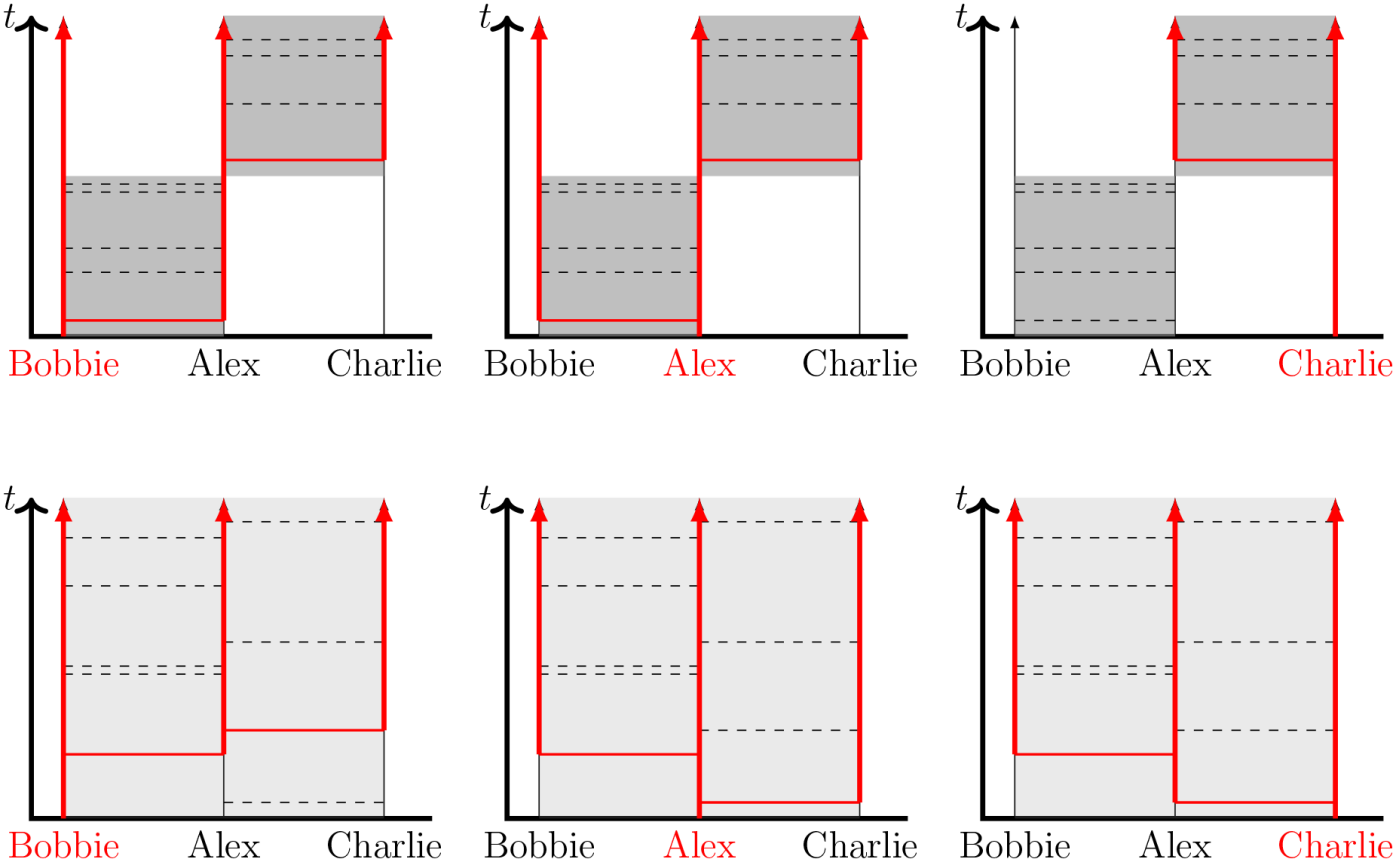
Sample scenarios comparing serially monogamous (top) and concurrent (bottom) relationships. Shaded regions denote the existence of a partnership between “Alex” and either “Bobbie” or “Charlie”, with darker shading representing a partnership having a higher transmission rate. Dashed lines denote transmission events within the relationship that would cause infection if one individual were infected and the other susceptible. Vertical red lines denote time at which an individual is infected, and horizontal red lines denote successful transmissions. In the concurrent case, the transmission events occur at exactly the same times, but the transmission could occur in either partnership. Thus the interaction rate within each partnership is half that of the serial case. Concurrency provides additional transmission routes and tends to speed up onwards transmission. In the left panels Bobbie begins infected, in the cenral panels Alex begins infected, and in the right panels Charlie begins infected.

It is worth exploring a simplified scenario to illustrate the key mechanisms by which concurrency can affect disease transmission as well as some of the subtleties, which make ultimate impact less obvious and make observational study design difficult. Consider an individual “Alex” who has two partners “Bobbie” and “Charlie” over a period of one year. Two potential partnership arrangements are shown in Fig 1. In the serial case (top of Fig 1), Alex’s partnership with Bobbie lasts for six months and is replaced by a six-month partnership with Charlie. In the concurrent case (bottom of Fig 1), Alex’s partnerships with Bobbie and Charlie overlap completely. During each partnership, there are occasional “transmission events”, or interactions between the individuals that would cause infection if one individual were infected and the other susceptible. If a transmission event occurs between an infected and a susceptible individual, then it is “successful” and the susceptible individual becomes infected. In Fig 1, and throughout this paper, we assume that the rate of potentially infectious interactions per individual is the same. Thus in a population of only serial partnerships, the rate of interactions per partnership is twice that in a population with exactly two overlapping partnerships per individual. The partnership duration is scaled so that the expected number of transmission events per partnership is the same. This allows us to ensure that in our comparisons the only change is the level of concurrency, and our results are not conflated with the effect of increased interactions per individual or per partnership. This is illustrated in Fig 1 by halving the temporal density of transmission events and doubling the partnership duration in the concurrent case.

First consider direct transmission to Alex from Bobbie or Charlie. Regardless of the partnership arrangement or which partner is initially infected, the overall risk of infection to Alex is the same. However, the timing of infection differs. If Bobbie is initially infected, then Alex tends to be infected earlier in a serial partnership than a concurrent one (because of the focused relationship). Conversely, if Charlie is initially infected, then Alex becomes infected earlier in a concurrent partnership (because of the delay forced by partnership timing in the serial case).

Now consider indirect transmission between Bobbie and Charlie via Alex. Significant differences in both the risk and timing of infection now exist depending on partnership arrangement. If Charlie is initially infected, then a transmission chain from Charlie to Alex to Bobbie is only possible in the concurrent case. If Bobbie is initially infected, then a transmission chain from Bobbie to Alex to Charlie will happen faster in the concurrent case because there is no built-in delay for partnership change. In turn this would allow Charlie to begin transmitting to other partners earlier. However, the probability of a Bobbie to Alex to Charlie transmission chain is slightly reduced because some of the interactions between Alex and Charlie will already have happened by the time Alex is infected (an example is given in the Discussion section later).

Finally, consider the result if Alex enters the partnerships already infected. The outcomes are the same regardless of whether the populations have concurrency, but concurrency tends to reduce the average time to transmission because the partnerships can start sooner. Unless Bobbie or Charlie also have concurrent relationships, this has no population-scale impact.

Thus we anticipate that concurrency will increase the spread of disease through two key mechanisms: by allowing the disease to trace transmission routes faster and by providing additional transmission routes. The subtleties described above can limit the extent of its effect. Whether one individual has concurrent relationships only affects the outcome of a particular partnership if both partners are susceptible at the beginning of their partnership and at least one becomes infected during it. Furthermore, the identical risk of infection to Alex regardless of partnership arrangement in Fig 1 illustrates that the risk of concurrency is to partners of the individual with concurrent relationships rather than to the individual with concurrent relationships.

### Modeling approaches

Unfortunately, measuring or predicting the magnitude of the impact of concurrency has been difficult. Concurrency is difficult to directly measure. Even when it is identified, observational studies comparing an individual with serial relationships to an individual with overlapping relationships within a given sample population will not test for the effects of concurrency. Instead the study would need to compare their partners, which is more difficult.

Modeling studies have a different set of challenges. Models are usually either stochastic agent-based simulation [which we will call “stochastic simulations” or simply “simulations”] or equation-based [which we will call “analytic models”]. Stochastic simulation of concurrency is often difficult because of inherent difficulties in identifying which of many parameters governs an outcome as well as computational limitations on the populations considered. Analytic models in contrast have difficulties because the standard well-mixed population assumption of analytic models precludes the existence of concurrent relationships. There is a need for analytic modeling that avoids this assumption.

Because analytic models have not existed for populations with concurrent relationships, most modeling investigations of concurrency have used stochastic simulation. Many are reviewed in [14].

Recent work on analytic models has shown how they can be used to incorporate some partnership structure [16–18]. The work of [19–21] in particular used analytic models to investigate how concurrency alters the epidemic threshold. The very recent work of [22] provides a renewal equation from which dynamics of a model (similar to the one we present below) can be calculated. The model of [23] also provides a dynamic prediction, but it makes simplifying assumptions about the independence of partner status, partnership age, and partner age. Where correlations build up this can lead to erroneous predictions.

Recent work [24–28] developed an “Edge-Based Compartmental Modeling” (EBCM) approach which leads to differential equation models with only a handful of equations. The models exactly predict the large population dynamics of SI and SIR disease spread in static random networks [29, 30]. The approach has been generalized to networks with changing partnerships, but still assuming a closed population [27]. Because the HIV epidemic developed over decades, it is not appropriate to ignore individuals entering and leaving the at-risk population as they age. Further, if we ignore “birth” and “death” for an SI disease in a closed population eventually the disease reaches the full population. Nevertheless, this provides a starting point from which we might be able to develop a tractable model that does capture “birth” and “death”.

We adapt the EBCM approach to accommodate “births” and “deaths” representing entry into and exit from the at-risk population. We show that the resulting equations accurately predict the outcome of simulations in the large population limit, and our primary focus is on using the model to investigate the role that concurrency can play in the spread of a “Susceptible—Infected” (SI) disease such as HIV.

Our goals in this paper are:

1. To demonstrate an analytic model for disease spread in a dynamic population with concurrency and show that it exactly predicts simulated dynamics in the large population limit.
2. To use the model to identify important regimes under which concurrency does or does not have an important effect and to understand the underlying mechanism by which this occurs in the model.
3. To explore what features of these underlying mechanisms would need to be preserved in real-world scenarios in order for concurrency to have (or not have) a major impact on population-scale outcomes.

This paper is not intended to be an authoritative statement about the role of concurrent relationships in Africa, rather we hope that an improved understanding of the underlying mechanisms will improve the quality of the discussion.

## Materials and methods

In this section we introduce our stochastic population and disease model, state the governing equations for the large population limit, and briefly outline their derivation. The full derivation is given in the Supporting Information (S1 File).

### Population/Disease assumptions

We assume discrete time. This assumption is made to simplify the simulations we use to validate the analytic model. The continuous time version of the analytic model is presented in the SI.

**Fig 2:**
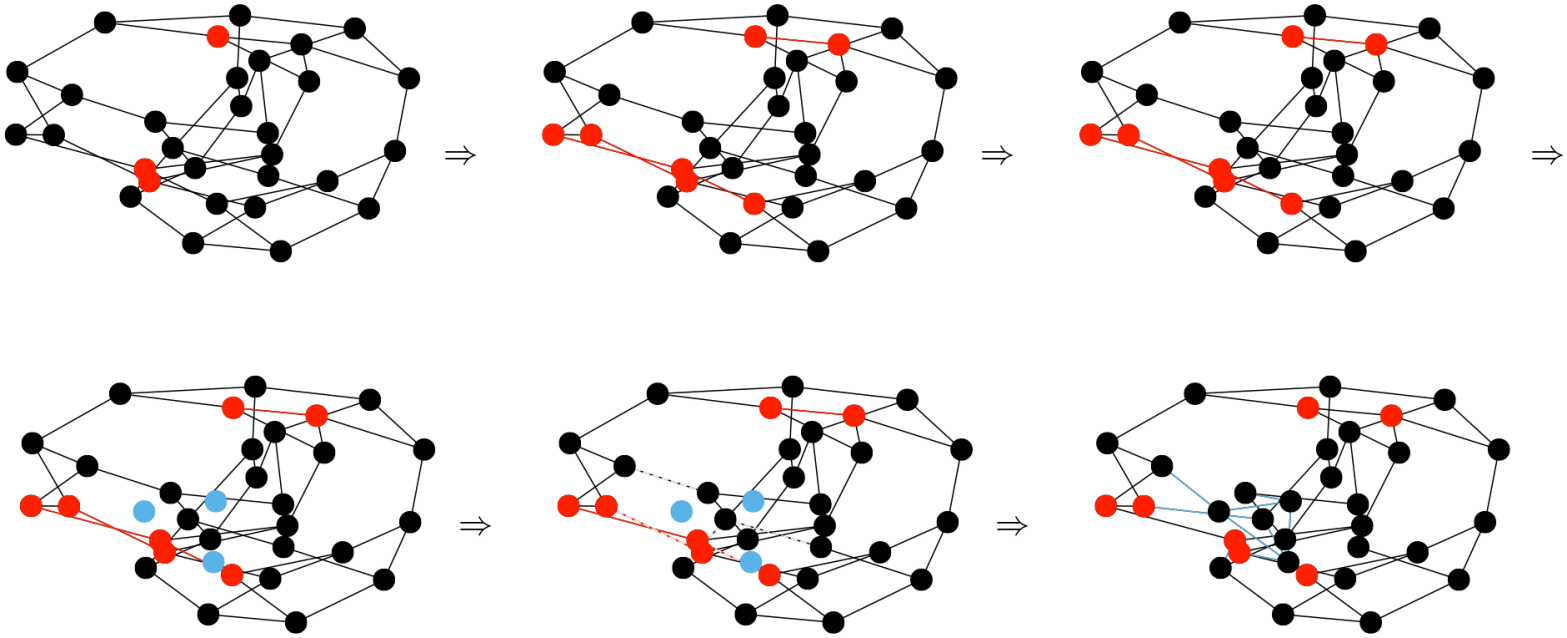
Sequence of events in each time step. We begin with a network with some infected individuals (red). Then infected individuals transmit to some partners (red edges). Then some individuals leave the population (white). Other individuals are born (blue). Then some partnerships break (dashed). Finally new partnerships are created so that the new individuals, the individuals whose partners left, and the individuals whose partnerships broke all return to their target number of partners. The sequence of events then repeats.

We initialize the network as a configuration model network [31]. In each time step actions occur in the following specific order (illustrated in Fig 2):

1. Each partnership connecting a susceptible to an infected individual transmits infection with probability *τ*.
2. Individuals leave the population independently with probability *μ*, freeing their partners to form a replacement partnership in step 5.
3. Next *μN* new individuals arrive where *N* is the imposed average population size. Each new individual’s “target degree”, or desired number of partners, *k* is assigned from the imposed degree distribution with probability *P*(*k*). The individual is given *k* free “stubs” or half-edges which will pair with other stubs to form partnerships.
4. Each existing partnership ends independently with probability *η*, freeing up the two stubs involved.
5. Free stubs are paired together at random until all individuals reach their target number of partners. If two stubs are chosen that come from the same individual or would duplicate an existing or a just-terminated partnership, they are left unpaired until the next time step (in the large population limit, this has a negligible impact).

This process is then repeated for the next time step. These assumptions are similar to those of [19, 20]. Python code implementing these steps is provided as a supplement (S2 File). We will present an analytic model that captures the large-population deterministic limit of these assumptions.

For the corresponding simulations, we choose the time step of the discrete-time framework to balance competing interests. We need a small time step so that *μ*, *η*, and *τ* are small (at leading order they are proportional to the time step), otherwise the arbitrary order of events impacts outcomes. However, for too small of a time step (and also in the continuous-time limit) few partnerships end in a time step. This makes it difficult for nodes to immediately find new partners. As the population size increases this becomes less of a problem so smaller time-steps become feasible. However, the computational effort becomes greater.

#### Governing equations

We give an overview of the derivation of the deterministic equations governing the susceptible and infected fractions. These equations are based on the “Edge-based Compartmental Modeling” approach of [18, 24, 26, 27].

Tables 1 and 2 summarize the parameters and variables of the model respectively. The model parameters are the initial fraction infected *ρ*, the per-time-step death probability *μ*, number of births *b*, transmission probability *τ*, and partnership change probability *η*. In addition to time *t*, the key independent variables are the age of an individual and the age of a partnership. The age of an individual gives some information about that individual’s status (a recent entrant is generally less likely to be infected than one who has been in the population for a while). In addition the age of a partnership gives information about the age of the partner (a partnership cannot be “older” than the partner). In calculating the risk an individual has from its partners, we need to account for the probability the partner has a given status. This depends on the age of the partner, which in turn depends on the age of the partnership, which itself is dependent on the age of the individual. To sort out the dependencies, the individual age and the partnership age are needed as independent variables. The resulting equations are low-dimensional, but are significantly more involved than even the dynamic network models presented in [27].

**Table 1:** The parameters for our simulations and equations. The last four are derived from the previous parameters.

Parameter	Description
*k*	The “degree”, or number of partners, an individual has (fixed in time).
*P*(*k*)	The probability a random individual has degree *k*.
*μ*	The probability a random individual will leave the population in a given time step.
*τ*	The transmission probability in a time step.
*η*	The probability a partnership will end in a time step.
*ρ*	The proportion of the population randomly infected at *t* = 0.
*b*	The number of individuals entering the population each time step (assumed to be constant).
*ψ*(*x*)	$\sum_{k=0}^{\infty }P\left(k\right)x^{k}$: The probability generating function of the degree distribution.
*p*_*b*_	1 − (1 − *μ*) (1 − *η*): The probability that a test individual’s partnership will end (break) because either the partnership ends naturally (rate *η*), or the partner leaves the population (rate *μ*). It does not include the possibility of the test individual leaving.
*P*_*e*_	$\frac{\left(1-\mu )(\eta +\mu -\eta \mu \right)}{(1-\mu )(\eta +\mu -\eta \mu )+\mu }$: The probability that a newly formed partnership will be with a previously existing individual.
*N*	*b*/*μ*: The average population size.

**Table 2:** The variables for our equations.

Variable	Description
*t*	Time
*u*	The test individual
*a*_*u*_	The age of test individual *u*, measured so that *a*_*u*_ = 0 in the first time step after *u* is born.
*a*_*e*_	Age of an partnership of interest, measured so that *a*_*e*_ = 0 in the first time step after the partnerships forms.
*S*(*t*)	The proportion of the population that is susceptible at time *t*, equivalently the probability a randomly selected individual is susceptible at time *t*, or equivalently the probability a test individual is susceptible at time *t*.
*I*(*t*)	1 − *S*(*t*): The proportion infected at time *t*.
*s*(*t*, *a*_*u*_)	The probability a test individual of age *a*_*u*_ is susceptible at time *t*.
Θ(*t*, *a*_*u*_)	The probability a stub belonging to *u* has not transmitted infection to it from a partner by the start of time step *t*.
Φ_*S*_(*t*, *a*_*u*_)	As for Θ (no partner has transmitted to *u* through the stub), but with the additional requirement that at the start of time step *t* the partner is susceptible.
Φ_*I*_(*t*, *a*_*u*_)	As for Θ (no partner has transmitted to *u* through the stub), but with the additional requirement that at the start of time step *t* the partner is infected.
*ϕ*_*S*_(*t*, *a*_*u*_, *a*_*e*_)	The probability that a stub belonging to an age *a*_*u*_ individual has not transmitted infection to it by time *t*, is connected to a susceptible partner, and the current partnership (or “edge”) has age *a*_*e*_.
χ(*t*, *a*_*e*_)	The probability that an age *a*_*e*_ partnership (or “edge”) of a test individual connects to a susceptible individual.

We seek the susceptible and infected fractions of the population *S* and *I*. We outline a simplified derivation ignoring some details of the initial condition and assuming the population has equilibrium size *N* = *b*/*μ*. The probability that a random individual *u* has age *a*_*u*_ is *μ*(1 − *μ*)^*a*_*u*_^ because the proportion born in any time step is *μ* (it must balance the proportion that die) and the probability of surviving *a*_*u*_ time steps is (1 − *μ*)^*a*_*u*_^. The probability that a random individual of age *a*_*u*_ and with *k*_*u*_ partners is susceptible is Θ(*t*, *a*_*u*_)^*k*_*u*_^ where Θ(*t*, *a*_*u*_) is the probability a random partner (or its predecessors along the same stub) has not transmitted to *u*. Thus the probability a random individual is susceptible is

$$\begin{array}{c} S\left(t\right)=\mu \sum_{a_{u}=0}^{\infty }({\left(1-\mu \right)}^{a_{u}}\sum_{k}P\left(k\right)\Theta {\left(t,a_{u}\right)}^{k}) \end{array}$$

Introducing the probability generating function *ψ*(*x*) = ∑_*k*_ *P*(*k*)*x*^*k*^, gives *s*(*t*, *a*_*u*_) = *ψ*(Θ(*t*, *a*_*u*_)) is the probability an age *a*_*u*_ individual is susceptible. This expression then simplifies to *S*(*t*) = *μ*∑(1 − *μ*)^*a*_*u*_^ *s*(*t*, *a*_*u*_). The probability a random individual is infected is *I*(*t*) = 1 − *S*(*t*).

We now derive an equation for Θ by noting that Θ(*t*, *a*_*u*_) = Θ(*t* − 1, *a*_*u*_ − 1) − *τ*Φ_*I*_(*t* − 1, *a*_*u*_ − 1) where Φ_*I*_ is the probability that the partner is infected and neither it nor any predecessor has transmitted to *u*. We find Φ_*I*_ by first solving for Φ_*S*_, the probability that the partner (and predecessors) have not transmitted and the partner is susceptible, and using Φ_*I*_ = Θ − Φ_*S*_. The derivation for Φ_*S*_ is given in the SI. It is similar to the derivation of *S*, but requires additional handling of the possible ages of the partner because the age distribution of the partners is different for partnerships of different ages.

Once we incorporate the initial condition and the full details of deriving Φ_*S*_, the governing equations are

$$\begin{array}{cc} S(t) & =\mu \sum_{a_{u}=0}^{\infty }{\left(1-\mu \right)}^{a_{u}}s\left(t,a_{u}\right)\\ s(t,a_{u}) & =\{\begin{array}{cc} \psi (\Theta \left(t,a_{u}\right)) & a_{u}<t\\ \left(1-\rho )\psi (\Theta (t,t)\right) & a_{u}\geq t \end{array}\\ I(t) & =1-S(t)\\ \Theta (t,0) & =1\\ \Theta (0,a_{u}) & =1\\ \Theta (t,a_{u}) & =\Theta \left(t-1,a_{u}-1\right)-\tau \Phi_{I}\left(t-1,a_{u}-1\right)\;t,a_{u}\geq 1\\ \Phi_{I}\left(t,a_{u}\right) & =\Theta \left(t,a_{u}\right)-\Phi_{S}\left(t,a_{u}\right)\\ \Phi_{S}\left(t,a_{u}\right) & ={\left(1-p_{b}\right)}^{a_{u}}\phi_{S}\left(t,a_{u},a_{u}\right)+p_{b}\sum_{a_{e}=0}^{a_{u}-1}{\left(1-p_{b}\right)}^{a_{e}}\phi_{S}\left(t,a_{u},a_{e}\right)\\ \phi_{S}\left(t,a_{u},a_{e}\right) & =\Theta \left(t-a_{e},a_{u}-a_{e}\right)\chi \left(t,a_{e}\right)\\ \chi (t,a_{e}) & =\{\begin{array}{ll} \left(1-\rho \right)\frac{\psi^{\prime }\left(\Theta \left(t,t\right)\right)}{\langle K\rangle } & a_{e}\geq t\\ \left(1-P_{e}\right)\frac{\psi^{\prime }\left(\Theta \left(t,a_{e}\right)\right)}{\langle K\rangle } & \\ \;+P_{e}\mu \sum_{a_{v}=a_{e}+1}^{t-1}{\left(1-\mu \right)}^{a_{v}-a_{e}-1}\Theta \left(t-a_{e},a_{v}-a_{e}\right)\frac{\psi^{\prime }\left(\Theta \left(t,a_{v}\right)\right)}{\langle K\rangle } & a_{e}<t\\ \;+P_{e}\left(1-\rho \right)\Theta \left(t-a_{e},t-a_{e}\right)\frac{\psi^{\prime }\left(\Theta \left(t,t\right)\right)}{\langle K\rangle }{\left(1-\mu \right)}^{t-a_{e}-1} & \end{array} \end{array}$$

We provide a full derivation of this model and give a continuous-time differential equations version in the Supporting Information (S1 File).

#### Specific modeled population

Although the equations allow different individuals to have a different number of partners, except where specifically noted we focus on populations in which all individuals have the same number of partners. In particular, this eliminates the need to consider how per-partnership transmission rates may depend on the individual’s number of partners. This allows us to focus on concurrency without the effect of some individuals having more frequent sexual activity than others.

So for our comparisons, *ψ*(*x*) = *x*^*k*^, 〈*K*〉 = *k* for some fixed value *k*. As a base case, we consider serial monogamy where each individual has a single partner (*k* = 1). Transmission occurs in a time-step with probability *τ*_1_ and partnerships end with probability *η*_1_. Individuals leave the population with probability *μ*.

We compare this with homogeneous populations having concurrency. We assume that for different values of *k* the populations are arranged such that the cumulative number of partners an individual has over a long period of time is the same. So populations with smaller *k* must have faster turnover. This implies that *η* = *η*_1_/*k* so that the *k* partnerships each lasts *k* times as long. Similarly we assume that the expected number of transmissions an infected individual would cause in a time step is the same. This requires *τ* = *τ*_1_/*k*.

#### Advantages of an analytic model

There are a number of important benefits to having an analytic model (even if it can only be solved numerically) as opposed to relying on stochastic simulation. In general, an analytic model allows us to gain much more insight into a system because the mathematical relationships that emerge can often be interpreted as an interaction between different physical effects from which we understand how system behavior emerges. In contrast, with stochastic simulation it is difficult to extract those mathematical relationships.

We highlight several practical advantages of analytic models.

- If our goal is to predict the large-population behavior, a single stochastic simulation may take much longer and use much more memory than a numerical solution of the analytic equations: even in the simplest well-mixed homogeneous population, we must have enough individuals in the simulation to accurately capture the average, while the numerical solution only needs to track the average. In our comparisons the numerical solution can run hundreds of times faster than a large stochastic simulation, and use several orders of magnitude less memory.
- If we want to see how the equilibrium size changes as parameters change, we can simulate to equilibrium, then change the parameters a bit and simulate further watching the system relax to the new equilibrium. With the numerical equations, we can do something similar, but once we identify the equilibria for multiple parameter values, we can then give good estimates of the equilibrium variables at new parameters. This can be fed into the numerical solution as an initial condition, allowing for dramatically faster convergence. We cannot use the same sort of extrapolation to find good initial conditions for a new stochastic simulation.
- The analytic model allows for the application of mathematical tools that are difficult to adapt to simulations. For example, the numerical solution of the differential equations version can use adaptive step size and other numerical techniques. We can look for properties of equilibria by assuming that the analytic variables do not change. We can look for relationships between the parameters which determine when epidemic growth is possible.

## Results

In this section we briefly compare the analytic model with simulations to show that the analytic model gives accurate predictions. We then use the analytic model to investigate the impact of concurrency on the endemic equilibrium and on the early growth rates. The impact of concurrency saturates in both cases, with the impact on the endemic equilibrium saturating at lower levels of concurrency than the impact on early growth.

### Accuracy of the analytic model

**Fig 3:**
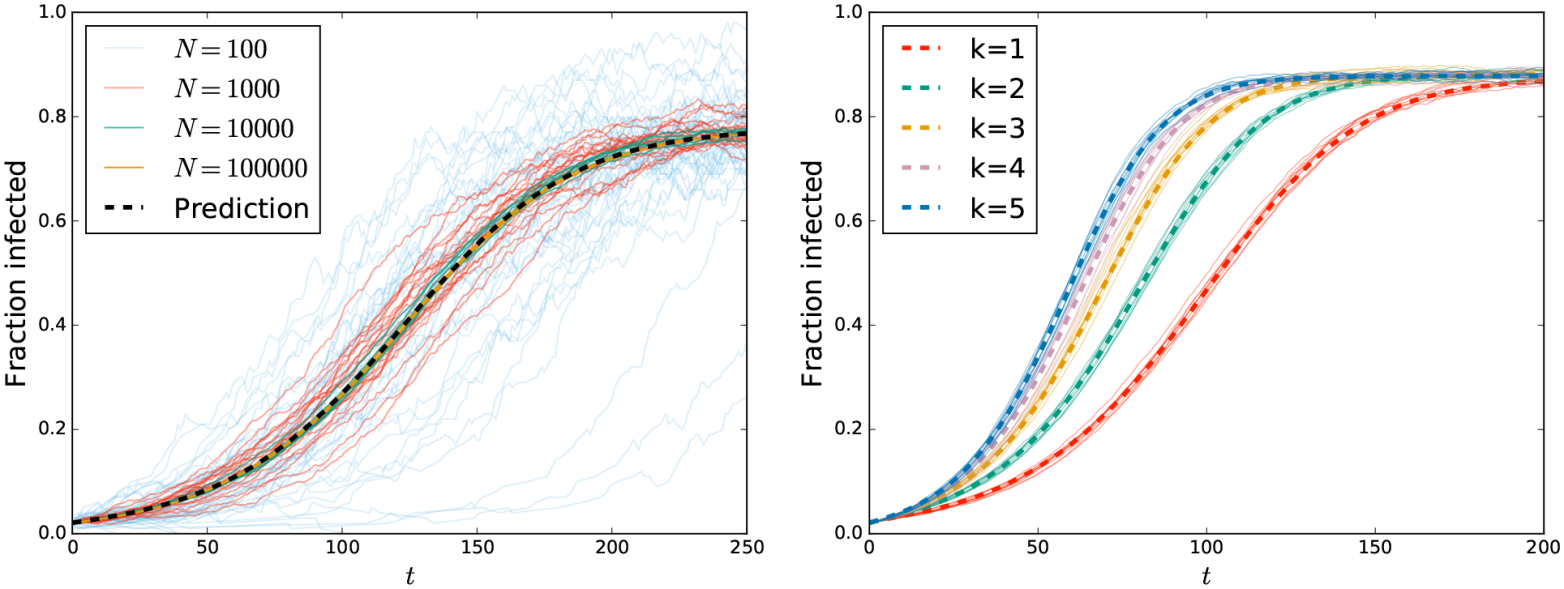
Comparison of analytic predictions and stochastic simulations. (Left) Stochastic epidemic simulations in populations of average size *N* = 10^2^, 10^3^, 10^4^ and 10^5^ with *k* = 3, *η* = 0.2/3, *τ* = 0.05/3, *ρ* = 0.02, and *μ* = 0.01. As *N* increases, the simulations converge to the analytic prediction. (Right) Comparison of analytic predictions and simulations for different values of *k*, with *η*_1_ = 0.1, *τ*_1_ = 0.1, *ρ* = 0.02, *μ* = 0.01 and average population size *N* = 10^4^ with *τ* = *τ*_1_/*k* and *η* = *η*_1_/*k*. We find excellent agreement between predictions (thick dashed curves) and stochastic simulations (thin solid curves).

**Fig 4:**
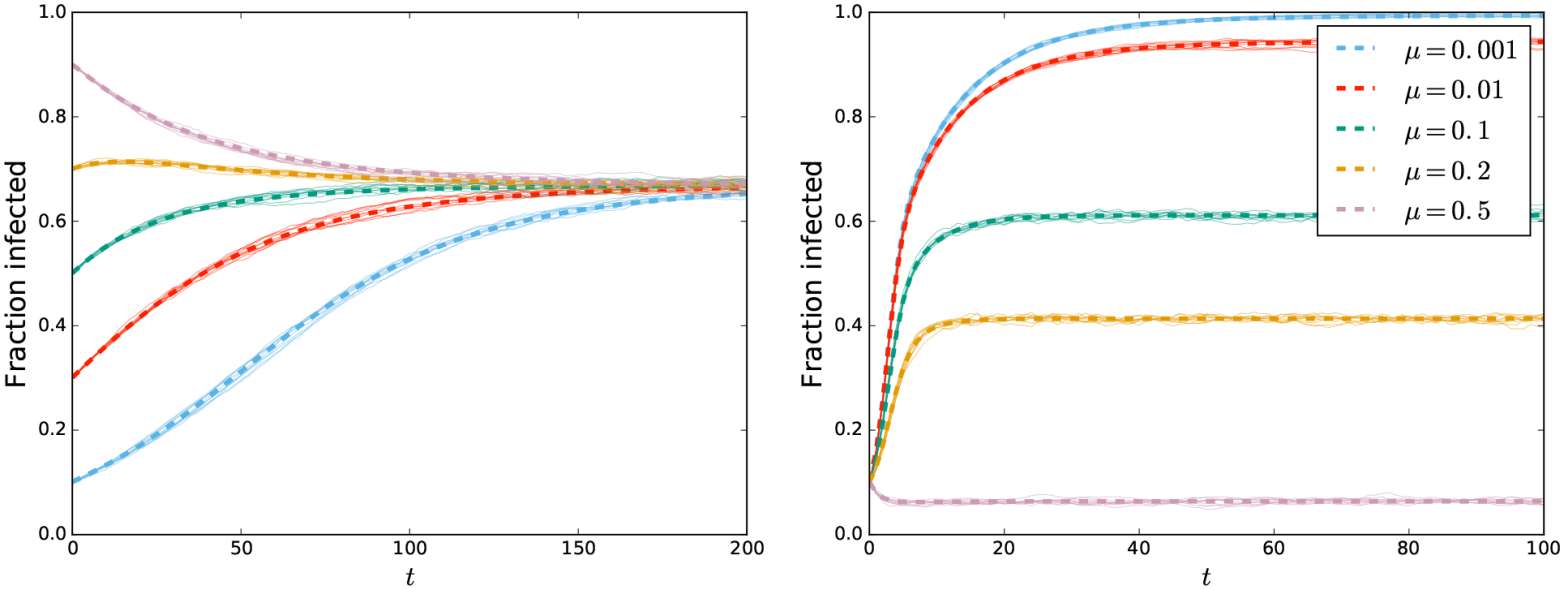
Comparison of dynamics from analytic predictions and stochastic simulations in populations with heterogeneous degree. (Left) Disease spread in populations with average size *N* = 10^4^ and degree probabilities *P*(2) = *P*(7) = 1/2. The parameters are *τ* = 0.01, *η* = 0.005, and *μ* = 0.01. The initial fraction infected *ρ* varies. (Right) Disease spread in populations with average size *N* = 10^4^ and degree probabilities *P*(1) = 1/2, *P*(10) = 1/3, and *P*(20) = 1/6. The parameter *μ* varies between populations. The remaining parameters are *η* = 0.05 and *τ* = 0.1. In both plots the dashed curves are analytic predictions and thin solid curves are stochastic simulations.

Figs 3 and 4 compare solutions of the analytic model with stochastically simulated epidemics across a range of conditions. The left plot of Fig 3 shows that as the population size increases, stochastic simulations converge to the predicted dynamics. The right plot considers populations in which all individuals have *k* partners for *k* = 1, 2, 3, 4, and 5 with *τ* = *τ*_1_/*k* and *η* = *η*_1_/*k* for *η*_1_ = *τ*_1_ = 0.1 and *μ* = 0.01. Simulations and predictions are a good match for different values of *k*.

Fig 4 looks at disease spread in populations with heterogeneous degrees, again showing excellent agreement between stochastic simulations and analytic predictions.

Our main conclusion from Figs 3 and 4 is that the equations accurately predict the large-*N* dynamics of simulations regardless of the parameters used.

### Further observations

From the left plot in Fig 4 we infer that for a given population, the initial proportion infected *ρ* does not influence the final state. At small *ρ*, the early dynamics are dominated by new infections to high- and low-degree nodes. At large *ρ* they are dominated by removal of infected high- and low-degree nodes. Interestingly, we see that for some intermediate values of *ρ* the prevalence initially grows and then decays. At these intermediate values, initially more high-degree nodes are being infected than are leaving while more low-degree nodes are leaving than being infected. Thus the two sets of nodes have opposing impacts on the dynamics. In this case, the growth in high-degree infections is initially the dominant effect, but it saturates while many low-degree infected individuals are still being removed.

From the right plot of Fig 4, we infer that increasing the population turnover rate decreases the proportion infected. This is not particularly surprising as it implies that infected individuals leave the population sooner, having had less opportunity to cause further infections.

### Impact of concurrency

**Fig 5:**
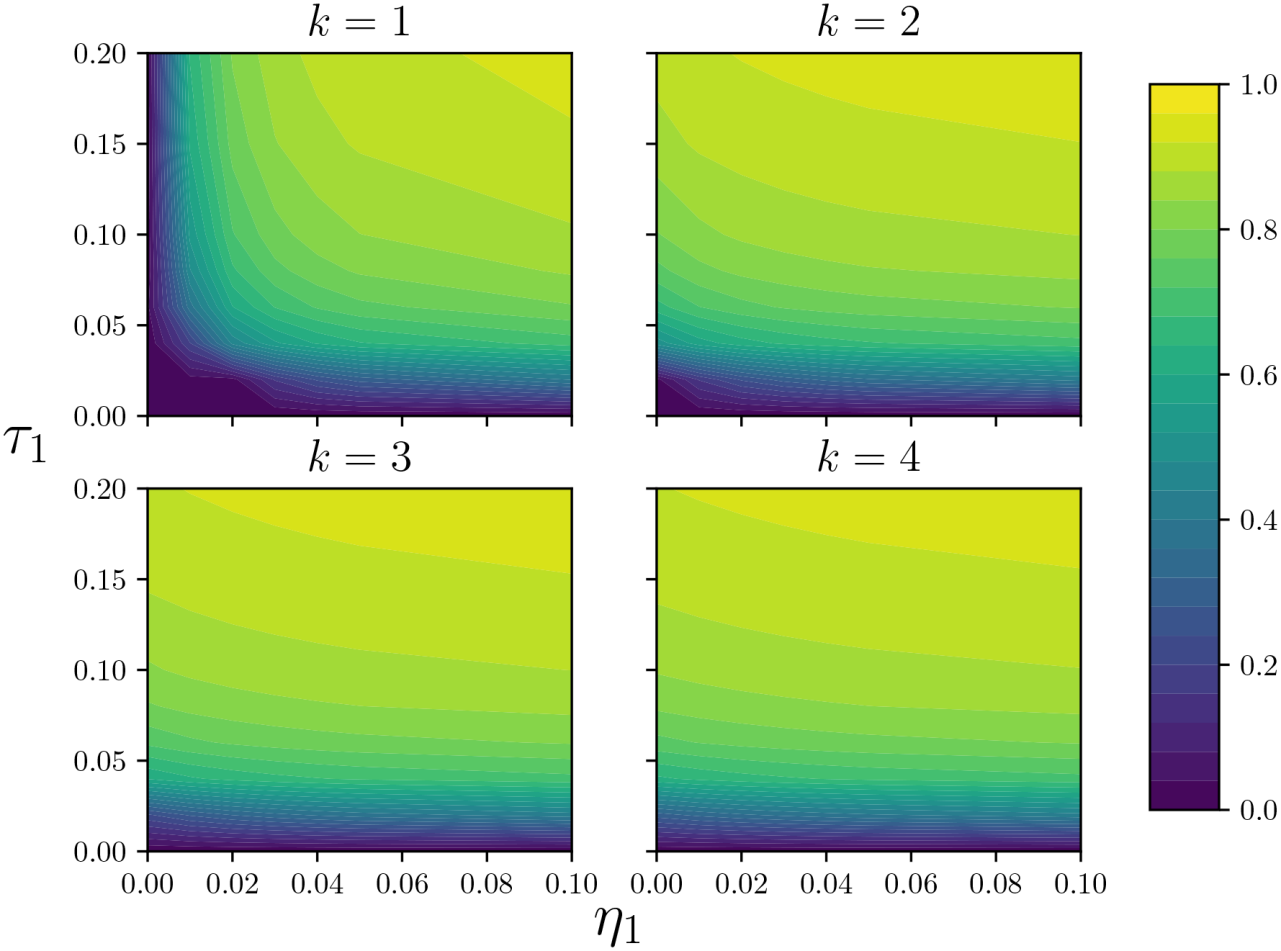
Comparison of equilibrium sizes for different values of *k*. The equilibrium fraction infected for *μ* = 0.01 and different *η*_1_ and *τ*_1_. We use the same axes *η*_1_ and *τ*_1_, taking *η* = *η*_1_/*k* and *τ* = *τ*_1_/*k*. (Top left) *k* = 1, (Top right) *k* = 2, (Bottom left) *k* = 3, and (Bottom right) *k* = 4. As *k* increases, the figures converge: the effect of concurrency on the equilibrium size quickly saturates.

**Fig 6:**
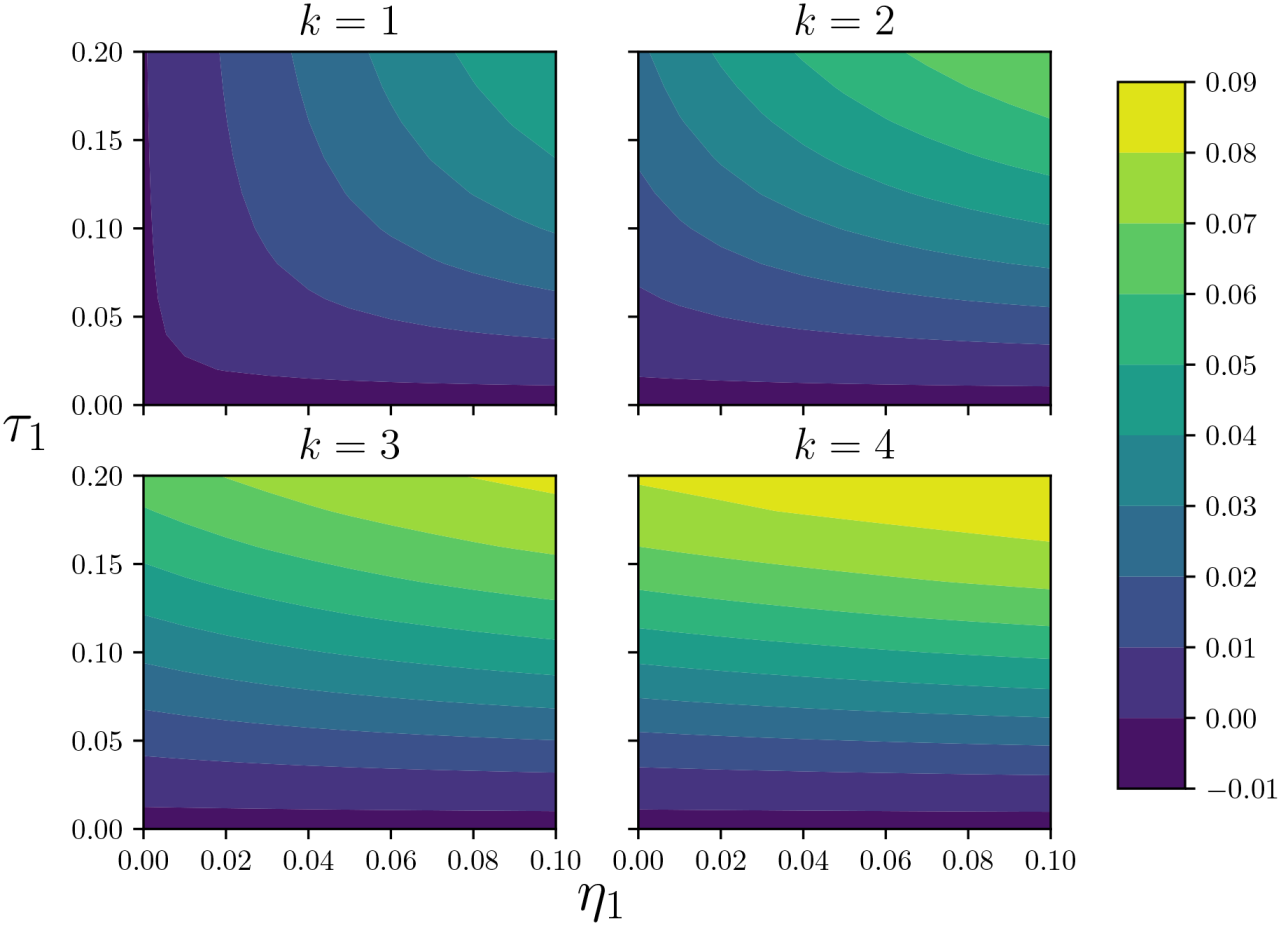
Comparison of early growth with different levels of concurrency. Contour plots of Δ*I*/*I* once the dynamics have entered the early exponential phase. (Top left) *k* = 1, (Top right) *k* = 2, (Bottom left) *k* = 3, and (Bottom right) *k* = 4. Although eventually the change saturates with larger *k*, it does not saturate as quickly as the equilibrium size does.

We now specifically explore the model predictions as we change the amount of concurrency. The right-hand plot of Fig 3 provides a special case of our more generic results. Interestingly we see that the equilibrium level does not vary significantly as concurrency increases, but the early growth rate does. Figs 5 and 6 show how the equilibrium infection levels and early growth rates change as *k* changes for a range of values of *η*_1_ and *τ*_1_.

Fig 5 shows that the impact of concurrency on the equilibrium epidemic size can be significant, but that the effect of increasing *k* may saturate quickly. The impact of concurrency is greatest if the partnership duration is long (*η*_1_ small). For much of parameter space, Fig 5 suggests that once a little concurrency is present, increased concurrency has little further effect on the equilibrium size.

In Fig 6, we demonstrate that in our model concurrency has a larger effect on the growth of the epidemic than on the equilibrium size. Although the impact of increasing concurrency on the early growth eventually saturates, it does so at larger *k* than for equilibrium size.

Figs 5 and 6 both show that saturation occurs soonest at lower transmission rates and higher partnership turnover rates, where the probability of multiple transmissions in a partnership is small.

## Discussion

We now discuss mechanisms whereby the impact of concurrency saturates, explore the implications for intervention design, and list caveats because of important effects neglected in our model.

We designed our comparisons so that, for different *k*, the number of partners an individual has over a long period of time and the total number of interactions within each partnership are the same. Thus we know that the effects we observe are not explained by within-population heterogeneity in degree, within-population heterogeneity in sexual activity rates, between-population differences in typical life-time number of partners, or between-population differences in the number of transmissions an individual causes per time step. All of these effects have been removed. Each population is homogeneous and they differ only in the number of concurrent partnerships.

To understand why concurrency does or does not matter in the different cases we take the perspective of the disease, observing transmission events and their outcomes. If concurrency has a significant impact on the population-scale spread of disease, it must be possible to infer the existence of concurrency by exploring the population structure in the same way the disease spreads. So we ask ourselves, “how easily can the disease measure the concurrency?”

### Low transmission rates/fast partnership turnover

In our model, we observed that concurrency has a reduced effect at lower transmission rate or if the partnership turnover rate is large. We now investigate why this is which will help us identify whether these observations should hold in the real world.

Looking back at Fig 1, we see that if we ignore the shading that designates when a partnership is in existence or not, it is still possible to infer which cases correspond to concurrent relationships by looking at the dashed lines that represent potential transmission events (that is interactions that would cause infection if the recipient were susceptible). The most obvious sign of concurrency is that at least one transmission event in one partnership lies between sequential transmission events in the other partnership.

**Fig 7:**
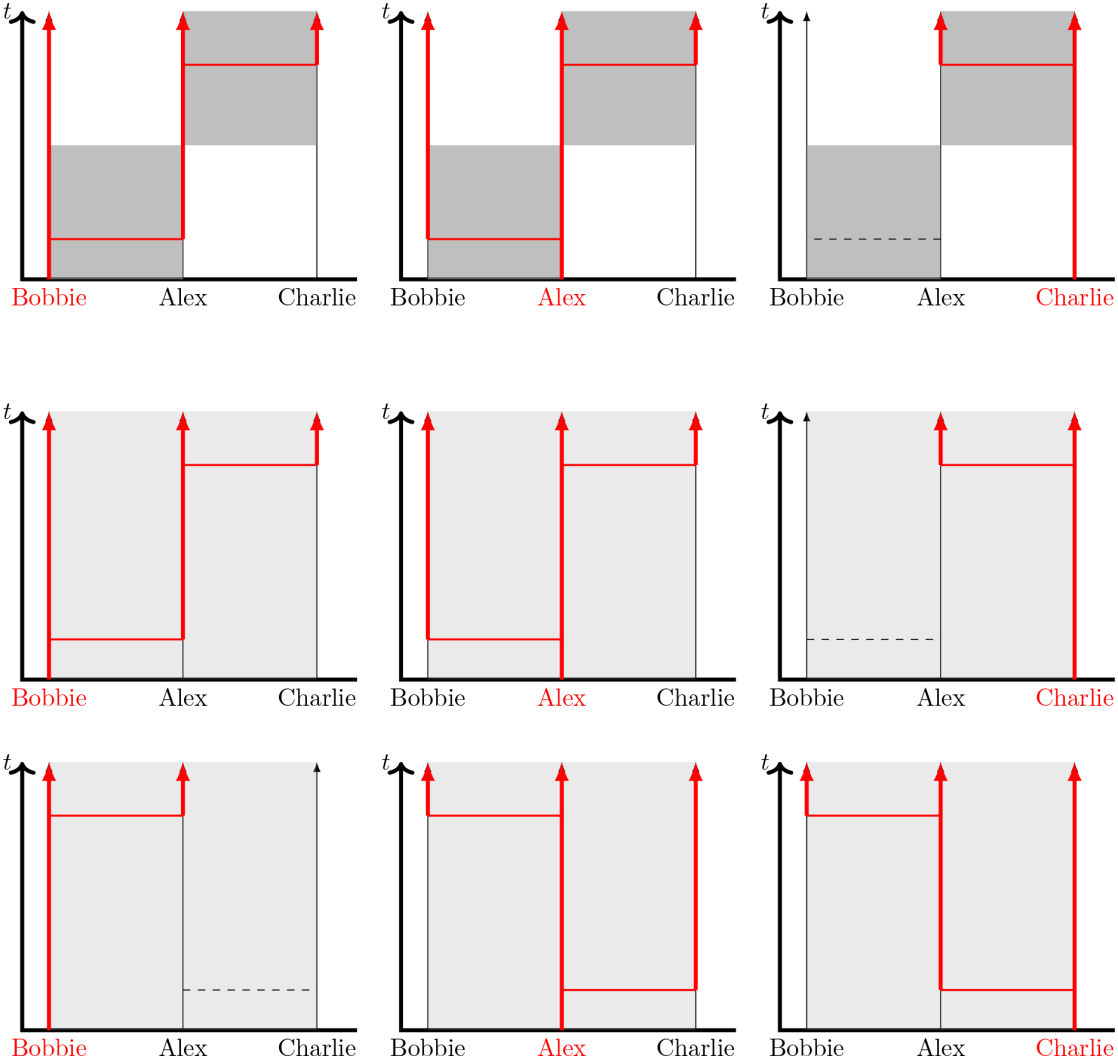
Scenarios for low transmission rates. (top) Serial monogamy case in which each partnership transmits once. (middle and bottom) Concurrent partnership case in which each partnership transmits once, with the order of transmission events differing in the two rows. Without the shading to denote the partnerships, it would not be possible to infer which cases exhibit concurrency, so we expect similar population-scale outcomes.

Investigating this closer in Fig 7, we see that indeed in the low transmission rate limit, we are unlikely to be able to distinguish between concurrent and serial partnerships by observing potential transmission events. Once almost every transmission event is to a different partner, the relevant detail is simply the interval between transmission events. The specific details of how long a partnership lasts, or how many concurrent partnerships exist are only marginally relevant. As long as the probability of transmitting twice to the same partner is negligible, the well-mixed population assumption would provide the same prediction (see also the “time-scale approximation” of [32] for more discussion of this). So the impact of concurrency when *τ*_1_/*η*_1_ ≪ 1 is negligible.

### Equilibrium sizes

We now turn to the equilibrium sizes which are predicted to be relatively insensitive to concurrency across much of parameter space. Before investigating this, we highlight that many real populations are likely to have low partnership turnover rates (*η*_1_), for which our model predicts the largest role of concurrency.

To explain the insensitivity of the equilibrium to concurrency, we note first that at equilibrium, an average infected individual causes one additional infection before leaving the population. Consider now a serially monogamous population and a population with concurrency, both at equilibrium, and consider a single individual who becomes infected.

As noted in the introduction, by itself, concurrency does not cause an infected individual to be more or less likely to transmit to a newly acquired partner. Rather, concurrency increases the probability an individual who is currently susceptible will later transmit to its currently susceptible partner.

So for concurrency to affect the equilibrium level, when an individual becomes infectious he or she must have a non-negligible probability of infecting an existing partner *at equilibrium*. At equilibrium, we only expect one successful transmission (some other transmissions will fail because the recipient is already infected). If the number of future partners is large compared to the number of current partners, we would expect that the one transmission is much more likely to go to a future partner than a current partner.

Put another way, if the risk to a susceptible individual from forming a new partnership with an already infected individual is much higher than the risk that an existing susceptible partner will become infected, then concurrency will not be a significant factor.

### Transient growth

We previously observed that concurrency can play a significant role in the early spread of disease even in scenarios where it has little effect on the long-term equilibrium. In this section we first address why the effect of concurrency saturates. Then we look at why the saturation occurs at higher levels of concurrency than for the equilibrium infection rates. We note that for a given average number of transmissions, the early growth is higher if those transmissions are concentrated earlier in the infectious period [33].

Looking at the disease’s perspective, early in an epidemic with a highly infectious disease, when an individual with many partners becomes infected, there will be rapid successful transmissions to many different partners. In contrast, in the serial monogamy case, there will generally be a delay following a successful transmission because a partnership change is required before the next successful transmission: the partnership dynamics constrain the spread. Thus in this scenario concurrency is expected to have an important impact on the early rate of spread.

As concurrency increases, we are keeping the same number of transmissions per partnership, but only the first transmission in a partnership is successful. By increasing concurrency, a larger fraction of the early transmissions an individual causes are the first transmission of the given partnership. Increasing concurrency thus reduces the effect of local depletion of susceptibles. At high enough concurrency the infected individual does not need to replenish its susceptible partner supply by changing partners. At this point, further concurrency will have little impact.

To explain why the saturation of concurrency occurs at higher levels for the growth rate than for the equilibrium level, we note that for determining the equilibrium, what matters is how many infections an individual causes, but the timing is not significant. The early growth however depends not just on how many infections are caused, but also on how quickly those infections occur. So the equilibrium is most affected by the fact that concurrency can increase the number of transmission chains, while the early growth is also affected by the fact that concurrency increases the speed with which those transmission chains are traced. Additionally, in the early growth regime, the increased number of transmission chains has a larger effect than near equilibrium where a non-negligible fraction of those additional chains are blocked by existing infection.

### Implications for intervention design

A major source of controversy about designing interventions to reduce concurrency is that it necessarily takes resources away from other interventions, and so those who question the magnitude of its effect understandably question the wisdom of implementing interventions.

Concurrency reduction would reduce transmissions early in the infectious period. In contrast, many interventions under consideration require identifying infected individuals and reducing their probability of onwards transmission. As these interventions are scaled up, a larger proportion of transmissions will be from more recently infected individuals increasing the relative value of interventions preventing transmissions from recently infected individuals. Thus concurrency reducing interventions may be a good complement to other interventions or a good followup once these other interventions are widely implemented.

We additionally note that our model raises the possibility that concurrency may have played a role in the rapid growth of the HIV epidemic in some regions, but that the role of concurrency in determining the resulting level of infection may have saturated. Thus, now that the epidemic is well-established, moderate reductions in concurrency might not lead to a rapid decay in the epidemic. This raises the threshold required for concurrency reduction to be effective: for a wide range of parameters reducing concurrency from a high level to a moderate level has much less impact on the epidemic than reducing concurrency from a moderate level to a low level. Thus concurrency-reducing interventions in well-established epidemics may be most effective in lower-concurrency settings.

An important setting which is not investigated in our model is the case in which a significant fraction of the population does not engage in concurrency. For these individuals, we would expect concurrent relationships of their partners to be a major source of their infection risk. Thus an intervention which encourages non-concurrent individuals to ensure that they partners who are also non-concurrent may well be successful. Our model could be used to test this with minor modifications.

### Caveats

There are a number of caveats of our study that must be highlighted to avoid overinterpreting these results. Understanding these limitations and why they might arise gives guidance on when we should expect concurrency to be important.

- **Acute Phase**: Before mounting an immune response, an individual’s viral load is several orders of magnitude larger than after the immune response develops. During this early phase infectiousness is dramatically increased [34–38]. If the individual has multiple partnerships, then many more infections can happen in this phase than would be seen if the individual were only in contact with its infector [39].
- **Impact of future interventions** As “treatment as prevention” or other interventions are implemented, it is likely that later partners will be at lower risk because treatment will reduce an individual’s infectiousness. This will increase the role of transmissions occurring early in an infectious period, increasing the relative role of concurrency.
- **Heterogeneous degree**: Some people have many more partnerships than others [40]. They generally become infected sooner, and in turn transmit to more individuals. Even if many individuals do not engage in concurrent relationships, if there a few with many concurrent partners, the effects may still be present. This provides an opportunity to reduce disease transmission through an intervention that encourages those without concurrent relationships to ensure their partners also do not have concurrent relationships.
- **Temporal behavior changes**: If the disease dynamics are driven by individuals having periodic high-risk episodes between long-term relationships, then the assumptions of this model are invalid. To correct this, the model must be adapted to allow for periods of high risk behavior, for example when a partnership ends.
- **Age structure**: If there is age-structure in the contact patterns, different effects may be seen. For example, we might think of the younger cohort as a population which has not yet been invaded by infection. In this case, the results about early growth as the disease invades this subpopulation may be more relevant than our model predicts. Reducing concurrency could be expected to play an important role in slowing the invasion of this younger cohort.
- **Coital dilution**: We have assumed that the transmission rate scales such that individuals have effectively the same total number of sexual acts regardless of their number of partners. This dilutes the number of acts per partnership, and to address this we extended the partnerships. This allows us to isolate the effect of concurrency from the effect of frequency of sexual acts. However, if concurrent relationships are associated with more (or less) frequent sexual acts, then the conclusions we reach here may not be valid. To correct for this, we would need to appropriately weight the transmission rates based on the number of concurrent relationships each partner has [41].

## Conclusions

We have derived an analytic model which accurately reproduces simulated SI epidemics in a population with concurrent relationships and demographic turnover. We use this model to isolate the role of concurrency in the spread of a disease such as HIV.

Although the model is highly simplistic, it can be generalized to incorporate more realism, and it can be used to help us understand important features of the role of concurrency in HIV spread. We see first that the impact of concurrency on the equilibrium size of SI epidemics can saturate. Consequently interventions targeting concurrency may have little impact unless they come close to eliminating concurrent relationships. However, we see a more significant role for concurrency in determining the early growth rate. As concurrency increases, the early growth is increased, and the effect saturates at higher concurrency levels than for the epidemic size. Thus reducing concurrency is likely to have more impact on early growth than on the final equilibrium.

An important additional observation from our analysis is that we can make significant progress by focusing on the “disease-eye” view of the population [42]. Using this, we have been able to explain why the numerical predictions of the model follow from the assumptions, and also gain insight into conditions under which we could expect our predictions to be robust to real-world conditions.

Our model is intended as a framework for developing more realistic models. Our goal has been to provide this framework and clearly demonstrate that it is possible to use analytic models to explore disease spread in populations with concurrent relationships with demographic turnover. The predictions our model has provided are true for the simplistic assumptions made. More careful models will be needed to identify conditions under which interventions targeting concurrency will be effective. These models will need to incorporate additional effects such as the acute phase of infection and more realistic information about degree distributions and correlations.

Our modeling introduces new issues which have not previously been considered in the concurrency discussion. In particular, even if a population has significant concurrency and even if that concurrency played a major role in the establishment and growth of the HIV epidemic in some population, it is not guaranteed that concurrency plays an important role in the current levels of infection. Thus although concurrency may cause an epidemic to grow quickly to its equilibrium, it is not clear that once the population reaches equilibrium reducing concurrency would significantly affect the equilibrium.

Regardless of the magnitude of the current impact of concurrency, it is likely that interventions such as TasP will disproportionately reduce transmissions caused later in the infectious period compared to transmissions caused earlier in the infectious period. As this happens, the relative impact of concurrency will increase.

## Supporting information

S1 File A derivation of the governing equations. (PDF)

S2 File Python code implementing simulations. (PY)
