# Supplementary material for: Saturation effects and the concurrency hypothesis: Insights from an analytic model
Source: PLoS One. 2017 Nov 14;12(11):e0187938. doi: 10.1371/journal.pone.0187938 (PMC5685581; doi:10.1371/journal.pone.0187938)
Supplement: S1 File — (PDF) [file pone.0187938.s001.pdf]

Supporting information for

Saturation Effects and the Concurrency Hypothesis: Insights from an Analytic Model

Joel C. Miller[1*] Anja C. Slim[2,3]

1 Institute for Disease Modeling, Bellevue, WA, USA
2 School of Mathematical Sciences, Monash University, Clayton, VIC, Australia
3 School of Earth, Atmosphere, and the Environment, Monash University, Clayton, VIC, Australia

* joel.c.miller.research@gmail.com

Our primary goal in the Supporting Information is to derive the governing equations. Although in the main text we assume all individuals have the same number of concurrent partners, in our derivation here we allow different individuals within the same population to have a different number of concurrent partners as long as all partnerships have the same transmission probability and typical duration. These assumptions could be modified and a number of other complexities added to the model we develop here, but we do not attempt this now.

Stochastic population and disease model

We now describe the stochastic rules we assume govern the population and disease dynamics. We use a discrete-time model. We begin with the population dynamics in the absence of disease. At each time step, $N\mu$ individuals enter the population, and each individual has probability μ to independently leave the population. This leads to an equilibrium population size of N, but with variation around this value.

Each individual u has a constant number of partners k_u which is assigned independently to u when u enters the population. $P(k)$ gives the probability that $k_u = k$. We think of u as having k_u "stubs" (also called "binding sites" by [1, 2]). The stubs pair with stubs from other individuals to form partnerships. When a partnership ends the two newly freed stubs join with other free stubs to form new partnerships. We assume that individuals immediately replace their partners so that at the start of each time step all individuals have a full set of partners ([3] has discussion of how to include more complicated partnership dynamics.).

We are interested in the epidemic timescale, which is longer than the individual's active period. So we must include "birth" and "death" or equivalently immigration and emigration.

We begin with a fraction ρ of the population randomly infected. In each time step, multiple events can happen. Since the order of events can matter (a partnership cannot transmit after it ends), we provide a consistent order, shown in figure 2. First, infected individuals transmit to their susceptible partners independently with probability τ. Second, individuals may "die" (or leave the population) independently with probability μ. Third, μN new individuals are added to the population (so the average number present is N) and assigned stubs. Fourth, each remaining partnership breaks with

probability η. Finally, the unpaired stubs form new partnerships, subject to the constraint that old partnerships are not reformed and individuals do not join to themselves. In simulations, these constraints are occasionally not satisfied, in which case the corresponding individuals wait a time step before attempting to form new partnerships. In a large population, the impact of this failure is negligible, and for our analytic equations below, we can assume that they are satisfied.

Equation Derivation

We now derive the discrete-time equations presented in the main text as well as a continuous-time version. These equations govern the large-population limit of our model.

Preliminaries

It will be useful to define the function

$$\psi(x) = \sum_k P(k)x^k$$

to be the probability generating function of the degree distribution. It has some important properties: $\psi(1) = \sum P(k)1^k = 1$, $\psi'(1) = \sum kP(k)1^{k-1} = \langle K \rangle$ where $\langle \cdot \rangle$ denotes the mean of the random variable.

Our derivation is based on [3]. We review the concept of a "test individual" (effectively equivalent to the *cavity state* of [4]). We start with the assumption that the population-scale dynamics are deterministic in the large population limit. A direct consequence of this assumption is the observation that the probability a randomly selected individual has a given status equals the proportion of the population with that status.

Although in the asymptotic limit, they have the same value, calculating the probability a random individual has a given status turns out to be simpler than calculating the proportion of individuals in each state. This is because of a simplification that results from the observation that the probability a single randomly chosen individual u has a given status is not affected if we prevent u from infecting any other individuals (Although it is not necessary here, it may be helpful to recognize that that the assumption the stochastic process exhibits deterministic population-scale dynamics means that a change of out come for a vanishingly small fraction of events does not alter the population-scale dynamics.). If we prevent u from transmitting to its partners, then the status of its partners become independent of one another.

Guided by this, we define a *test individual* to be an individual u chosen uniformly at random from the population and prevented from transmitting infection. We have the following sequence of questions which have identical answers if the dynamics are deterministic: Given the initial proportion infected ρ,

1. What fraction of individuals are susceptible or infected at time t?
2. What is the probability a random individual is susceptible or infected at time t?
3. What is the probability a randomly chosen test individual is susceptible or infected at time t?

The first two equations have the same answer because we assume ρN is large enough that the dynamics may be treated as deterministic. The last two equations have the same answer because preventing a single individual u from transmitting does not affect

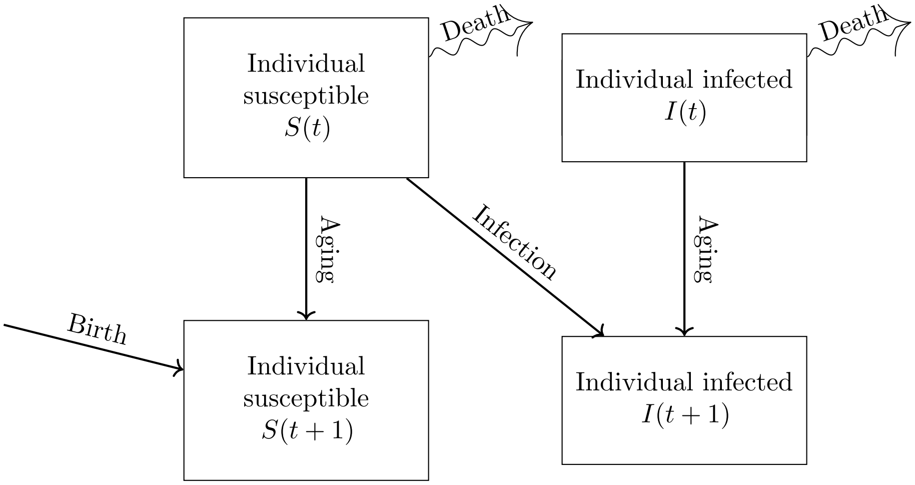

Fig S.1. Basic transitions of individuals from time t to $t+1$. At each time step, death occurs with probability μ. At population equilibrium, the proportion of the population that has age 0 is thus the number born divided by the total population, that is, $\mu = b/N$. The class S can be subdivided based on how long an individual has been in the population. Note that deaths are exactly balanced by births (at population equilibrium).

its probability of changing status (we highlight that we are *not* asking what proportion of nodes are in each state once u is prevented from transmitting).

We start our calculations with the goal of finding $S(t)$ and $I(t)$ using figure S.1. The processes such as death and aging and birth are relatively straightforward to model. However, the probability an individual becomes infected in a time step is not independent of the individual's age: For example, an individual who has been in the population longer will have a different distribution of partnerships from someone who has only recently joined. Consequently we must subdivide $S(t)$ based on individual age a_u. These subdivisions are shown in figure S.2. At equilibrium, the proportion of the population which is age 0 is μ (which equals b/N), and the probability that such an individual is susceptible is $s(t,0) = 1$. At each subsequent time step, these aging individuals are removed with probability μ, and so the proportion of the population with age a_u is $\mu(1-\mu)^{a_u}$. The probability that such an individual is susceptible is defined to be $s(t,a_u)$. To find $s(t,a_u)$, we turn to $\Theta(t,a_u)$, the probability that a stub belonging to an age a_u test individual u has never been involved in a transmission to u. Once we know that, then the probability a test individual of age a_u and k_u partners is susceptible at time t is $\Theta(t,a_u)^{k_u}$. Averaging this over the entire population of age a_u individuals the probability an age a_u individual is susceptible is

$$s(t,a_u) = \begin{cases} \psi(\Theta(t,a_u)) & a_u < t \\ (1-\rho)\psi(\Theta(t,a_u)) & a_u \geq t \end{cases}$$

where we recall $\sum_k P(k)x^k = \psi(x)$, and the $1-\rho$ factor in the second term accounts for the fact that the individual would be infected at $t=0$ with probability ρ.

The fraction susceptible is thus

$$S(t) = \mu \sum_{a_u=0}^{\infty} (1-\mu)^{a_u} s(t,a_u)$$

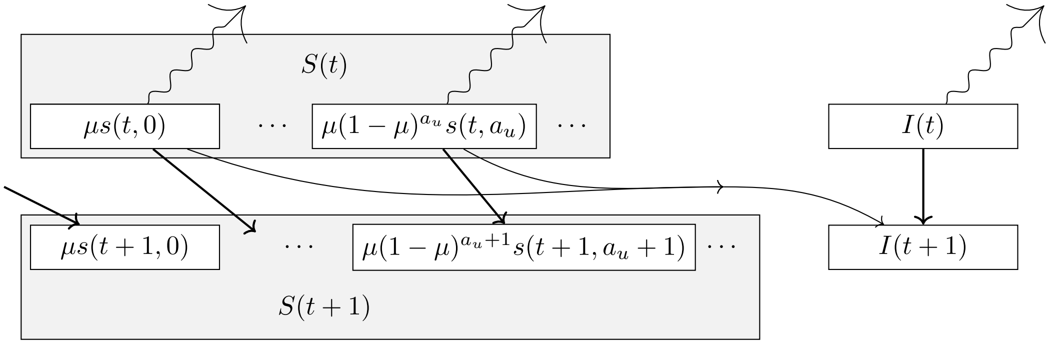

Fig S.2. The subdivisions of class S. The variable $s(t,a_u)$ denotes the probability that an age a_u individual is susceptible. Thus $s(t,0)=1$. The number of individuals in each age class is $\mu(1-\mu)^{a_u}$, representing the fact that a proportion μ are born in a given time step, and of these $(1-\mu)^{a_u}$ survive to step a_u. The arrows show the fluxes out of the compartment corresponding to susceptible individuals of age a_u. There is no need to subdivide the $I(t)$ compartment. Rather than calculating the flux along each arrow, we will derive explicit expressions for $s(t,a_u)$.

The probability of being infected is

$$I(t) = 1 - S(t)$$

We must now derive an expression for $s(t,a_u)$.

The focus of our calculations is on determining $\Theta(t,a_u)$. As a boundary condition we have

$$\Theta(t,0) = 1$$

stating that when an individual is first introduced, it has not yet received any infection. Similarly we have the initial condition

$$\Theta(0,a_u) = 1$$

as well, stating that prior to the disease introduction, no transmissions have occurred. Looking at figure S.3, we see that the change in Θ is from transmissions which occur with probability $\tau\Phi_I$. So the change in Θ in a time step is $-\tau\Phi_I$ where Φ_I is the probability that the stub has not previously brought infection to u and connects to an infected partner at the start of the time step. So we have

$$\Theta(t,a_u) = \Theta(t-1,a_u-1) - \tau\Phi_I(t-1,a_u-1)$$

However to do this calculation we require $\Phi_I(t,a_u)$ which is still unknown. We can shift our unknown from Φ_I to Φ_S (the probability the stub has not transmitted to u and currently connects to a susceptible partner) by using

$$\Phi_I = \Theta - \Phi_S\,.$$

As in calculating S, to calculate Φ_S, we turn it into a sum, following figure S.4. The probability that a partnership created when u joined still exists is $(1-p_b)^{a_u}$. The probability that a partnership has some smaller age a_e is $p_b(1-p_b)^{a_e}$. So

$$\Phi_S = (1-p_b)^{a_u}\phi_S(t,a_u,a_u) + p_b\sum_{a_e=0}^{a_u-1}(1-p_b)^{a_e}\phi_S(t,a_u,a_e)$$

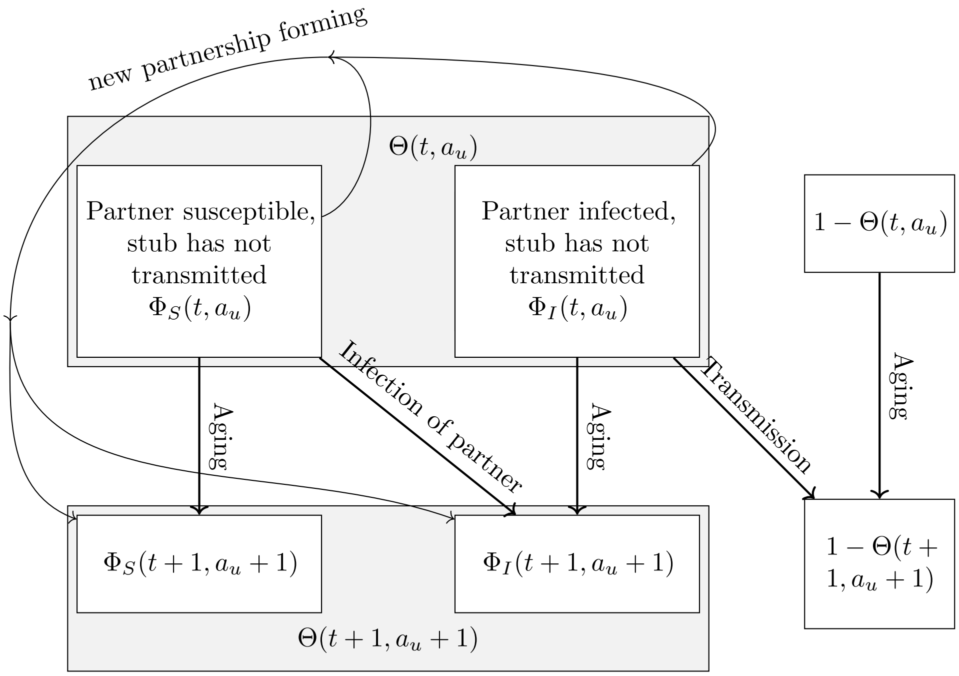

Fig S.3. Basic transitions of partnerships from time t to $t+1$ (from the perspective of an age a_u test individual who does not die at time t). At each time step the partnership ends with probability $\mu + \eta$. . The class Φ_S can be subdivided based on how long a partnership has existed.

where $\phi_S(t, a_u, a_e)$ is the probability that a stub belonging to an age a_u individual that is part of an age a_e partnership has not transmitted by time t and p_b is the probability that a stub is freed to find a new partnership (either by death of the partner, or termination of the partnership). The one term outside the sum represents the fact that when the individual first enters the population the stub definitely forms a partnership.

We now find $\phi_S(t, a_u, a_e)$. If the partnership formed when u was born ($a_e = a_u$) then this is simply the probability the partner v is susceptible given that v has an age a_u partnership with u, which we denote $\chi(t, a_u)$. However, if the partnership formed after u was born ($a_e < a_u$), then $\phi_S(t, a_u, a_e)$ is the probability $\Theta(t - a_e, a_u - a_e)$ that the stub was not responsible for transmitting infection to individual u prior to the current partnership forming times $\chi(t, a_e)$. As $\Theta(t - a_u, 0) = 1$ these coincide when $a_u = a_e$, so we can write

$$\phi_S(t, a_u, a_e) = \Theta(t - a_e, a_u - a_e)\chi(t, a_e)$$

We now find $\chi(t, a_e)$ similarly to $s(t, a_u)$. It is

$$\chi(t, a_e) = \sum_{A_v=a_e}^{\infty} P(a_v = A_v|a_e)P(v \text{ susceptible}|a_v = A_v)\,.$$

If $a_e \geq t$, then we know that v was born either when the disease was introduced or earlier. Thus no previous partnership could have transmitted to v. if we assume $a_v = A_v \geq a_e$, then the probability v is susceptible is the probability that it escaped infection when the disease was introduced $1 - \rho$ times the probability that it has not been infected by any other partners. Because of how v is selected (it is u's partner), v is

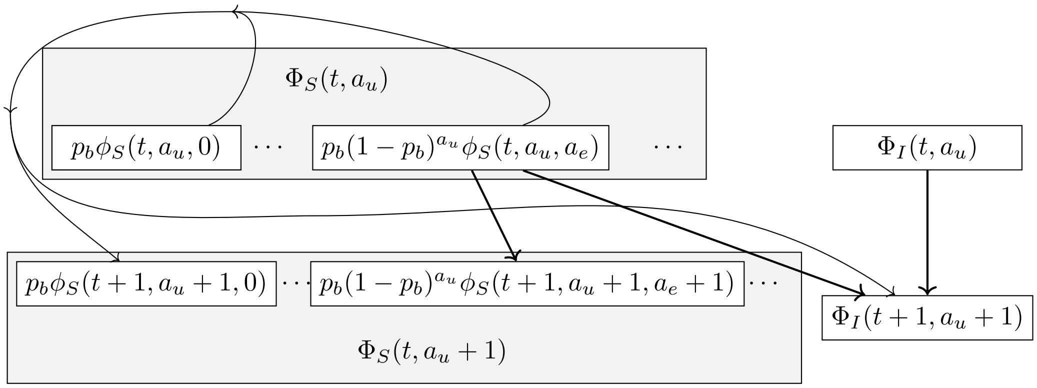

Fig S.4. The subdivisions of the Φ_S compartment. Arrows show the fluxes out of the subcompartment corresponding to partnerships of age a_e. Rather than calculating the fluxes along each arrow, we will explicitly calculate $\phi_S(t, a_u, a_e)$.

likely to have a higher degree than a randomly selected individual. The probability v has degree $k_v = k$ is $kP(k)/\langle K\rangle$. So the probability v is susceptible given A_v is $(1-\rho)\sum_k [kP(k)/\langle K\rangle]\Theta(t, A_v)^{k-1} = (1-\rho)\psi'(\Theta(t, A_v))/\langle K\rangle$. Thus for $a_e \geq t$ we have

$$\begin{aligned}\chi(t, a_e) &= \sum_{A_v=a_e}^{\infty} P(a_v = A_v|a_e)P(v \text{ susceptible}|a_v = A_v)\\ &= \sum_{a_v=a_e}^{\infty} \mu(1-\mu)^{a_v-a_e}(1-\rho)\frac{\psi'(\Theta(t, a_v))}{\langle K\rangle} \qquad a_e \geq t\end{aligned}$$

For $a_e < t$ there are three important cases to consider based on whether the partner was born before or at the same time that the partnership was formed and whether the partner was born before or after the disease was introduced.

- If the partnership formed when v was born then $A_v = a_e$ for which $P(a_v = a_e|a_e) = (1 - P_e)$ and $P(v \text{ susceptible}|a_v = a_e) = \sum_k [kP(k)/\langle K\rangle]\Theta(t, a_e)^{k-1} = \psi'(\Theta(t, a_e))/\langle K\rangle$, which measures the probability that another partner of v has not transmitted to v.
- If v was born before the partnership formed but after the disease was introduced then $a_e < A_v < t$ and $P(a_v = A_v|a_e) = P_e\mu(1-\mu)^{A_v-a_e-1}$. Although u has not transmitted to v, it is possible that a previous partner of v that was eventually replaced by u did. Thus the probability v is susceptible is $\Theta(t - a_e, A_v - a_e)\psi'(\Theta(t, A_v))$.
- If v was born before the disease was introduced, then $A_v \geq t$. We again have $P(a_v = A_v|a_e) = P_e\mu(1-\mu)^{A_v-a_e-1}$, but there is an extra factor of $1-\rho$ in the probability v is susceptible.
$P(v \text{ susceptible}|A_v) = (1-\rho)\Theta(t - a_e, A_v - a_e)\psi'(\Theta(t, A_v))$.

So for $a_e < t$ we have

$$\begin{aligned}
\chi(t, a_e) &= \sum_{A_v=a_e}^{\infty} P(a_v = A_v|a_e) P(v \text{ susceptible}|a_v) \\
&= P(a_v = a_e|a_e) P(v \text{ susceptible}|a_v = a_e) \\
&\quad + \sum_{v=a_e+1}^{t-1} P(a_v = A_v|a_e) P(v \text{ susceptible}|a_v = A_v) \\
&\quad + \sum_{A_v=t}^{\infty} P(a_v = A_v|a_e) P(v \text{ susceptible}|a_v = A_v) \\
&= (1 - P_e) \frac{\psi'(\Theta(t, a_e))}{\langle K \rangle} \\
&\quad + P_e \mu \sum_{a_v=a_e+1}^{t-1} (1-\mu)^{a_v - a_e - 1} \Theta(t - a_e, a_v - a_e) \frac{\psi'(\Theta(t, a_v))}{\langle K \rangle} \\
&\quad + P_e \mu (1-\rho) \sum_{a_v=t}^{\infty} (1-\mu)^{a_v - a_e - 1} \Theta(t - a_e, a_v - a_e) \frac{\psi'(\Theta(t, a_v))}{\langle K \rangle}
\end{aligned}$$

Simplification for $a_u > t$

We claim that the value of $\Theta(t, a_u)$ is the same for all $a_u \geq t$. This follows from the fact that at $t = 0$ all the values are 1. By inspecting the equations for the evolution of Θ, we see that if we assume $\Theta(t, a_u)$ is the same for all $a_u \geq t$, then the change in Θ is also the same. Thus we can assume $\Theta(t, a_u) = \Theta(t, t)$ if $a_u > t$. This argument would break down if partnership formation were affected by age differences.

Among the resulting simplifications is the observation that for $a_e \geq t$, the expression for $\chi(t, a_e)$ simplifies to $(1-\rho)\psi'(\Theta(t,t))/\langle K \rangle$.

Governing Equations

Our full system of equations becomes

$$
\begin{aligned}
S(t) &= \mu \sum_{a_u=0}^{\infty} (1-\mu)^{a_u} s(t,a_u) \\
s(t,a_u) &= \begin{cases} \psi(\Theta(t,a_u)) & a_u < t \\ (1-\rho)\psi(\Theta(t,t)) & a_u \geq t \end{cases} \\
I(t) &= 1 - S(t) \\
\Theta(t,0) &= 1 \\
\Theta(0,a_u) &= 1 \\
\Theta(t,a_u) &= \Theta(t-1,a_u-1) - \tau\Phi_I(t-1,a_u-1) \qquad t,a_u \geq 1 \\
\Phi_I(t,a_u) &= \Theta(t,a_u) - \Phi_S(t,a_u) \\
\Phi_S(t,a_u) &= (1-p_b)^{a_u}\phi_S(t,a_u,a_u) + p_b \sum_{a_e=0}^{a_u-1} (1-p_b)^{a_e}\phi_S(t,a_u,a_e) \\
\phi_S(t,a_u,a_e) &= \Theta(t-a_e,a_u-a_e)\chi(t,a_e) \\
\chi(t,a_e) &= \begin{cases} (1-\rho)\frac{\psi'(\Theta(t,t))}{\langle K\rangle} & a_e \geq t \\ \\ (1-P_e)\frac{\psi'(\Theta(t,a_e))}{\langle K\rangle} & \\ +P_e\mu\sum_{a_v=a_e+1}^{t-1}(1-\mu)^{a_v-a_e-1}\Theta(t-a_e,A_v-a_e)\frac{\psi'(\Theta(t,a_v))}{\langle K\rangle} & a_e < t \\ +P_e(1-\rho)\Theta(t-a_e,t-a_e)\frac{\psi'(\Theta(t,t))}{\langle K\rangle}(1-\mu)^{t-a_e-1} & \end{cases}
\end{aligned}
$$

We can derive a differential equations version of this by treating the time step as Δt rather than 1 and assuming that the event probabilities are all proportional to Δt. Then taking $\Delta t \to 0$ yields differential equations. We will use $\hat{\mu} = \lim_{\Delta t\to 0} \mu/\Delta t$ and similarly define other variables.

In the continuous time case, we find

$$
\begin{aligned}
S(t) &= \mu \int_0^\infty e^{-a_u \mu} s(t, a_u)\, \mathrm{d}a_u \\
s(t, a_u) &= \begin{cases} \psi(\Theta(t, a_u)) & a_u < t \\ (1-\rho)\psi(\Theta(t,t)) & a_u \geq t \end{cases} \\
I(t) &= 1 - S(t) \\
\Theta(t, 0) &= 1 \\
\Theta(0, a_u) &= 1 \\
\left(\frac{\partial}{\partial t} + \frac{\partial}{\partial a}\right)\Theta(t, a_u) &= -\tau \Phi_I(t, a_u) \\
\Phi_I(t, a_u) &= \Theta(t, a_u) - \Phi_S(t, a_u) \\
\Phi_S(t, a_u) &= e^{-p_b a_u} \phi_S(t, a_u, a_u) + p_b \int_0^{a_u} e^{-p_b a_v} \phi_S(t, a_u, a_e)\, \mathrm{d}a_e \\
\phi_S(t, a_u, a_e) &= \Theta(t - a_e, a_u - a_e)\chi(t, a_e) \\
\chi(t, a_e) &= \begin{cases} (1-\rho)\frac{\psi'(\Theta(t,t))}{\langle K \rangle} & a_e \geq t \\ \\ (1-P_e)\frac{\psi'(\Theta(t,a_e))}{\langle K \rangle} & \\ +P_e \mu \int_{a_e}^t e^{-\mu(A_v - a_e)} \Theta(t - a_e, A_v - a_e) \frac{\psi'(\Theta(t,A_v))}{\langle K \rangle}\, \mathrm{d}A_v & a_e < t \\ +P_e(1-\rho)\Theta(t - a_e, t - a_e)\frac{\psi'(\Theta(t,t))}{\langle K \rangle} e^{-\mu(t - a_e)} & \end{cases}
\end{aligned}
$$

The simplest numerical method to solve this system of equations would discretize by age and apply an Euler method, which corresponds to solving the discrete-time equations above.

References

1. Leung KY, Kretzschmar M, Diekmann O. Dynamic concurrent partnership networks incorporating demography. Theoretical population biology. 2012;82(3):229–239.

2. Leung KY, Kretzschmar M, Diekmann O. SI infection on a dynamic partnership network: characterization of R_0. Journal of mathematical biology. 2014;71(1):1–56.

3. Miller JC, Slim AC, Volz EM. Edge-Based Compartmental Modelling for Infectious Disease Spread. Journal of the Royal Society Interface. 2012;9(70):890–906.

4. Karrer B, Newman MEJ. Message passing approach for general epidemic models. Physical Review E. 2010;82:016101.
